# Transient imaging changes accompany ‘spinal ICANS’ following CAR T‐cell therapy for large B‐cell lymphoma in adults

**DOI:** 10.1111/bjh.70206

**Published:** 2025-10-13

**Authors:** Frederick W. Vonberg, Daniel Rice‐Wilson, Imran Malik, Stephen Keddie, Nikos Gorgoraptis, Shamzah Araf, Sunita Khanal, Silvia Montoto, Shenbagaram Kasivisvanathan, Ben Uttenthal, Katerina Panopoulou, Harpreet Hyare, Maeve O'Reilly, Aisling S. Carr, Claire Roddie

**Affiliations:** ^1^ Department of Neuromuscular Disease Institute of Neurology, Queen Square, University College London London United Kingdom; ^2^ Centre for Neuromuscular Diseases, National Hospital for Neurology and Neurosurgery University College London Hospitals London United Kingdom; ^3^ Department of Neurology Royal London Hospital, Barts Health NHS Trust London United Kingdom; ^4^ Department of Haematology University College London Hospitals London United Kingdom; ^5^ Department of Haemato‐oncology St. Bartholomew's Hospital, Barts Health NHS Trust London United Kingdom; ^6^ Department of Haematology Cambridge University Hospitals Cambridge United Kingdom; ^7^ Department of Radiology University College London Hospitals London United Kingdom; ^8^ Cancer Institute, University College London London United Kingdom

**Keywords:** CAR‐T, ICANS, myelopathy, paraparesis

To the Editor,

Neurotoxicity occurs in up to 64% of patients following chimeric antigen receptor T‐cell (CAR‐T) therapy.^1^ Recognised syndromes include immune effector cell‐associated neurotoxicity syndrome (ICANS),^2^ movement and neurocognitive treatment‐emergent adverse events (MNT)^3^ and tumour inflammation‐associated neurotoxicity (TIAN).^4^ As the use of CAR‐T expands into broader cancer and autoimmune conditions, rarer neurotoxic syndromes are increasingly recognised, including spinal cord deficits or myelopathy.^5^

To date, the global experience of spinal cord deficits associated with CAR‐T therapy for adult B‐cell cancers is summarised by Deschenes‐Simard et al.^5^ and highlights several important learning points.

First, of the 20 post‐CAR‐T myelopathy cases presented, CAR‐T was identified as the driver of the syndrome in 10/20 (50%) (following reasonable exclusion of alternative causes), and several shared clinical features were observed between patients. Briefly, patients were relatively young (average age 36.8 years; median age of US CAR‐T recipients ~60 years); all had received CD19‐targeted CAR‐T products, of which the majority (6/10) received axicabtagene ciloleucel (axi‐cel); and 9/10 patients developed myelopathy in the context of grade 4 (high‐grade) ICANS. Together, these features suggest a potential relationship between high‐grade cerebral ICANS (which can be severe in younger patients and in those receiving CD28z CAR‐T products^6^) and spinal manifestations of ICANS. Accordingly, very few cases of myelopathy have been reported in adults treated with 4‐1BBz CARs, which are broadly associated with less severe neurotoxicity.^6^

Second, 4/20 cases were not due to ICANS but to human herpesvirus (HHV)‐6 infection (2 patients), eosinophilic myelopathy (1 patient) and TIAN (1 patient).

Third, in the six remaining post‐CAR‐T myelopathy cases summarised by Deschenes‐Simard et al,^5^ there was diagnostic uncertainty. This speaks to the clinical challenge inherent in recognising novel, infrequent post‐CAR‐T clinical events in the absence of good diagnostic tests. While published data on diagnostic imaging for spinal ICANS are limited, there are some cases where longitudinally extensive spinal T2 hyperintensity has been described, with variable time to resolution (0.5–6 months^7^, ^8^, ^9^) or progression (2 months^10^).

Here, we present the largest series of acute, monophasic paraparesis or ‘spinal ICANS’ in adult patients post‐CD19 CAR‐T therapy for which high acuity clinical/paraclinical and brain/spinal imaging data are available for all patients, with a follow‐up of 3–19 months.

Four cases were identified from three specialist CAR‐T centres in the United Kingdom, and data were collected by retrospective case note review. All imaging was reviewed by a single neuroradiologist (HH). Retrospective ethics approval was obtained through the Integrated Research Application System (IRAS) with informed consent for the collection of minimally identifiable data as per European Society for Blood and Marrow Transplantation (EBMT) policy (IRAS ID: 285530). Serial imaging, cerebrospinal fluid (CSF) analysis and repeated neurological examination were performed for each patient.

Baseline patient characteristics, clinical examination, investigations including imaging and treatments administered are listed per patient in Table S1 along with clinical outcome at last follow‐up. The inpatient stay timeline per patient is illustrated in Figure 1. Full clinical case descriptions are provided in the Supporting Information Appendix.

**FIGURE 1:**
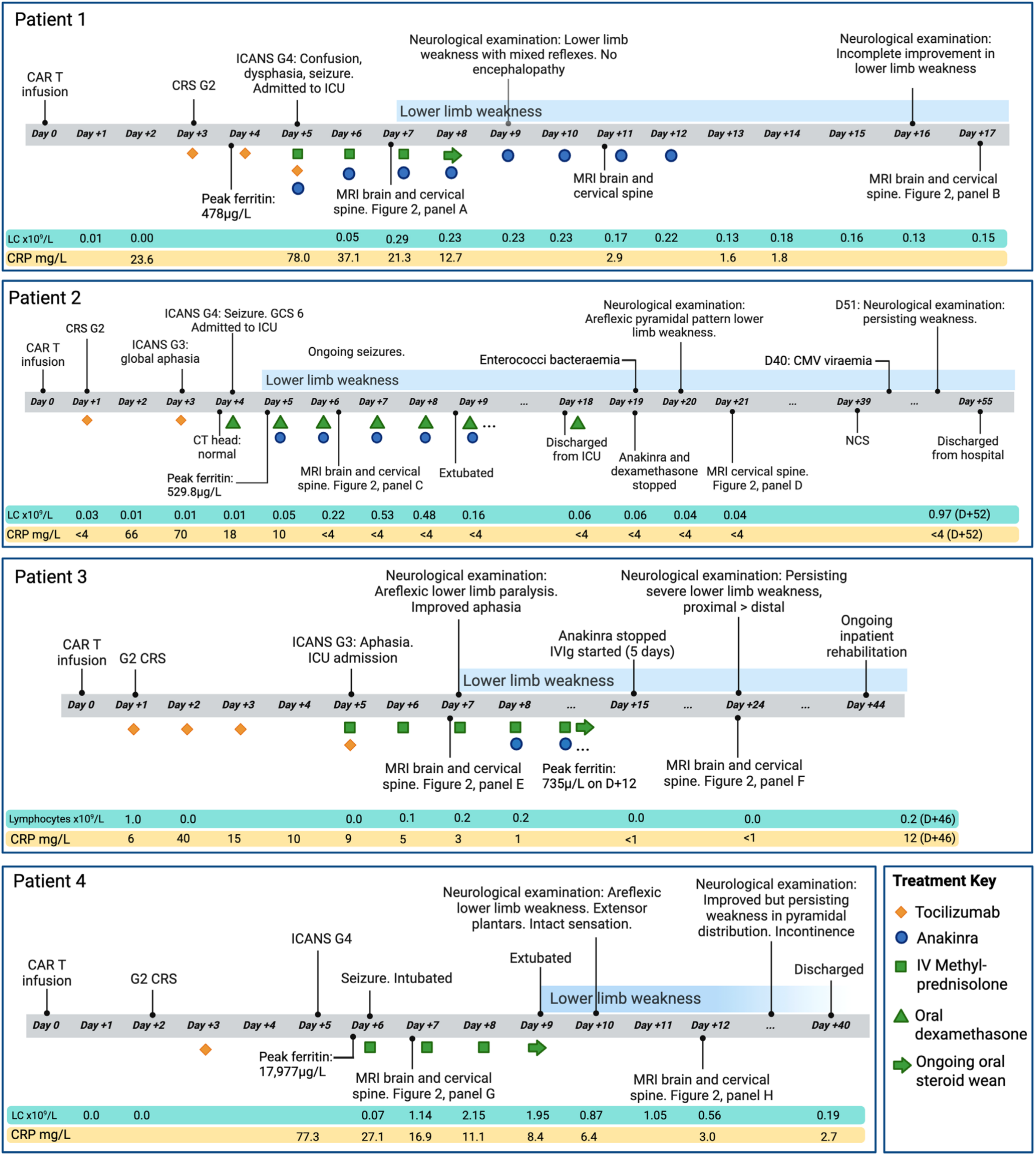
Timeline of clinical events for each of the four patients. Timing of MRIs are shown with reference to corresponding images in Figure 2. Timing of tocilizumab (gold diamonds), anakinra (blue circles), intravenous methylprednisolone (green squares) and oral dexamethasone (green triangles) are also shown. Steroid weaning following intravenous methylprednisolone is indicated with a green arrow. Daily blood results including CRP and lymphocyte count, alongside peak ferritin are illustrated. CAR‐T, chimeric antigen receptor T cells; CMV, cytomegalovirus; CRP, C‐reactive protein; CRS, cytokine release syndrome; CT, computed tomography; G, grade; GCS: Glasgow coma scale; ICANS, immune effector cell‐associated neurotoxicity syndrome; ICU, intensive care unit; IVMP, intravenous methylprednisolone; LC, lymphocytes; mg/L, milligrams per litre; MRI, magnetic resonance imaging; NCS, nerve conduction studies; μg/L, micrograms per litre.

All patients developed an acute onset, monophasic, diffuse myelopathic insult in the context of severe (grade 3–4) ICANS. Imaging showed diffuse T2/FLAIR hyperintensities throughout the cord on MRI (Figure 2) and albuminocytologic dissociation in the CSF, suggesting an inflammatory myelitis. In all cases, myelopathy was directly attributable to CAR‐T therapy, and we have therefore called the phenomenon ‘spinal ICANS’. Extensive investigations for other potential causes of spinal cord pathology were negative. Briefly, low CSF white cell counts with negative viral polymerase chain reaction (PCR) excluded an infective cause; the absence of malignant spinal involvement precluded TIAN^4^; the gradual improvement over weeks to months and the imaging findings were inconsistent with an ischaemic myelopathy. All patients demonstrated gradual clinical improvement following immune‐modulating treatment (Table S1; Figure 1). Resolution of imaging findings was more rapid than clinical improvement in all cases.

**FIGURE 2:**
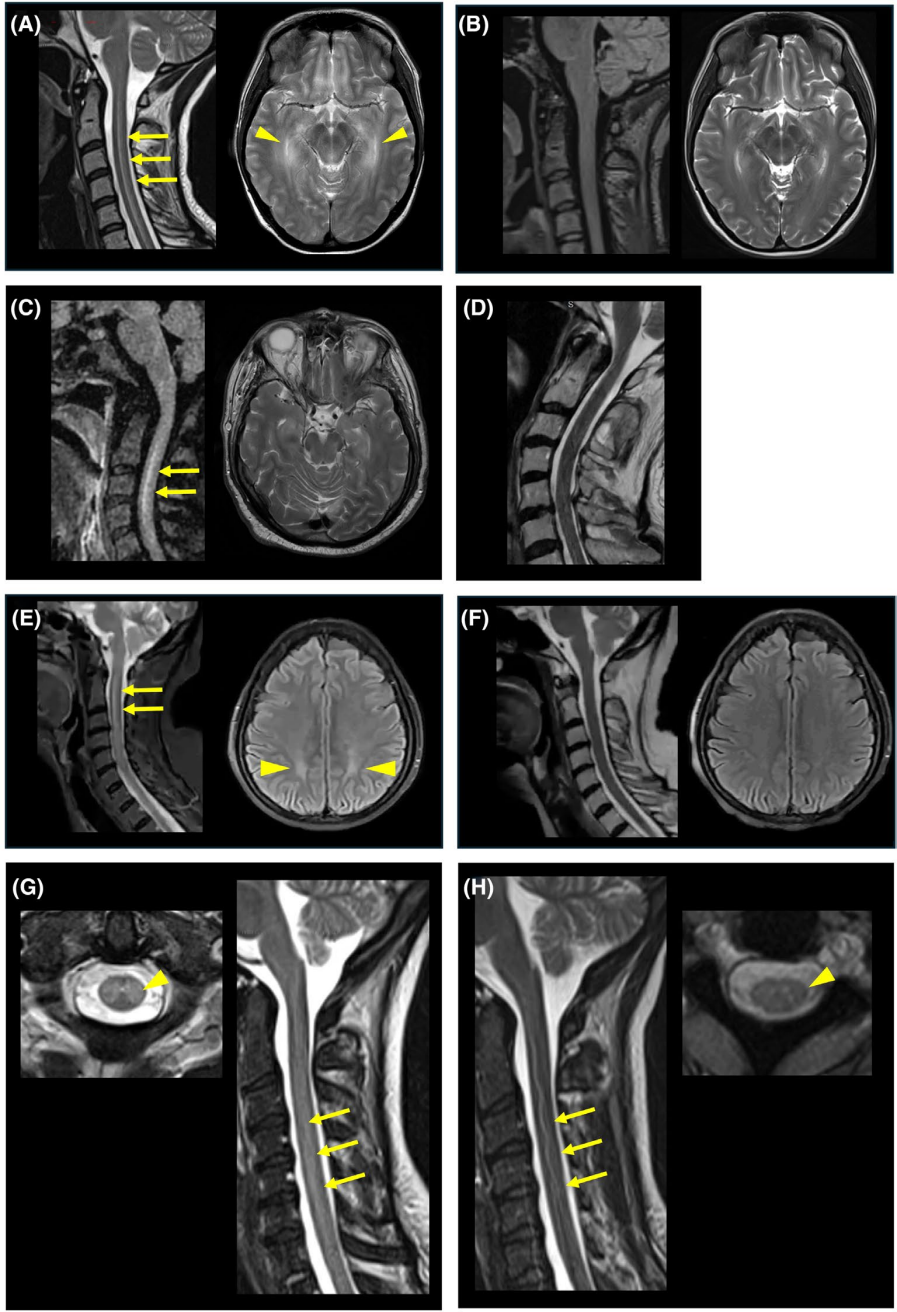
Magnetic resonance images for the four patients. Patient 1: T2‐weighted sagittal image of the cervical spine and axial image of the brain from day 7 (panel A) demonstrating widespread hyperintensity in the corticospinal tracts in the spine (arrows) and brain (arrow heads). Panel (B) shows T2/FLAIR image of the sagittal spine and T2‐weighted axial image of the brain from day 17, demonstrating slight improvement. Patient 2: T2/FLAIR sagittal image of the cervical spine and T2‐weighted axial image of the brain from day 6 (panel C) demonstrating longitudinally extensive signal change in the spine (arrows) and patchy hyperintensity in the brain. Panel (D) shows a sagittal T2‐weighted image of the cervical spine from day 21 demonstrating resolution of previous signal hyperintensity. Patient 3: T2‐weighted/FLAIR sagittal image of the cervical spine and axial image of the brain from day 7 (panel E) showing extensive, symmetrical signal hyperintensity throughout both posterior frontal, parietal and medial temporal lobes (arrow heads), with longitudinally extensive central cord hyperintensity in the cervical spine (arrows). Signal change extended throughout the length of the spine (not shown). The signal changes have completely resolved by day 24 (panel F). Patient 4: T2‐weighted axial and sagittal images of the cervical spine on day 7 (panel G) demonstrate hyperintensity within the white matter tracts (arrows and arrowhead). Repeat imaging on day 12 (panel H) shows marked regression, with some residual signal change. FLAIR, fluid‐attenuated inversion recovery.

The mechanism by which CAR‐T causes myelopathy is unknown. Consistent with other reports, we observed that affected patients were young and had received CD19‐targeted CARs with CD28z co‐stimulatory endodomains.^5^ Also in common with other published cases, myelopathy occurred exclusively in the context of severe ICANS^5^ and may reflect an extension into the cord of the inflammatory processes driving cerebral dysfunction in ICANS. This hypothesis is supported by the cases in our series where spinal imaging abnormalities were concurrent and continuous with the brain MRI changes typically described in ICANS. In our analysis, the prevalence of seizures, hypophosphataemia (commonly seen in the context of seizures) and raised CSF protein (reflecting blood–brain barrier [BBB] breakdown) were taken as additional indicators of ICANS severity beyond ‘Grade 4’ clinical scoring. Raised protein has previously been described in most,^9^, ^10^ but not in all^11^ cases of CAR‐T myelopathy where CSF is available and is also seen in ICANS without myelopathy. Further evidence for a systemic inflammatory aetiology includes raised C‐reactive protein and peak ferritin in all cases and illustrated per patient in Figure 1.

An underlying inflammatory aetiology for CAR‐T myelopathy is further supported by similarities with neuromyelitis spectrum disorder (NMSOD) and myelin oligodendrocyte (MOG) antibody‐associated disease (MOGAD), where longitudinally extensive transverse myelitis (LETM) over >3 vertebral segments is a hallmark feature. In these conditions, clinical presentations are sub‐acute in onset (days–weeks) and are characterised by severe functional deficits which improve with corticosteroids and plasma exchange. Patients usually have acellular CSF with elevated protein (albuminocytologic dissociation). Recovery is typically prolonged after effective immunosuppression of the inflammatory insult, likely reflecting gradual neuronal recovery (weeks to months) of the clinically eloquent, compact neuronal structures of the spinal cord.^12^

Diagnosing CAR‐T myelopathy can be challenging. The rarity of this neurological syndrome warrants appropriate investigation for alternative causes, but it may be an under‐recognised phenomenon. Difficulty in performing a detailed neurological examination in an individual with severe ICANS who may be intubated and unresponsive, coupled with the early resolution of spinal MRI features, may contribute to underdiagnosis. In our practice, we now actively screen for myelopathic deficits with daily neurological examination in high‐risk patients with severe ICANS, particularly in the context of seizures, and have a low threshold for MR imaging of the cervicothoracic spine.

If spinal ICANS is driven by the same mechanisms as cerebral ICANS, then early and assertive immunosuppression should adequately manage the underlying cytokine‐driven inflammatory pathology.^13^ However, recognising that prolonged high‐dose steroid is associated with increased mortality in ICANS,^14^ it may be prudent that corticosteroid treatment taper be guided by improvements in spinal MRI signal changes coupled with clinical improvements in cerebral ICANS, rather than waiting for the resolution of spinal deficits which can take weeks to months. Experience in other syndromes causing monophasic transverse myelitis suggests similarly slow clinical recovery, lagging behind resolution of the underlying inflammatory driver. This is thought to reflect slow neuronal repair rather than an ongoing active inflammatory insult.^12^

The asynchronous neurological recovery of cerebral versus spinal ICANS observed here accords with previous descriptions^8^, ^9^, ^10^ and may reflect a lack of functional redundancy in the spine where even small lesions and associated swelling/damage in this anatomically vulnerable site can lead to severe debilitation (the so‐called neuroanatomical‐functional paradox^15^). In contrast, the brain displays significant redundancy for cognitive functions, where ‘cognitive reserve’ is evidenced by patients with significant inflammatory lesion burden but relatively unimpaired cognitive function. It is also possible that the asynchronous recovery observed between central and spinal ICANS partly reflects the limited scope of existing neurocognitive assessment tools for ICANS, namely, the immune effector cell encephalopathy (ICE) score, to detect subtle cerebral deficits, such that we label patients as recovered from cerebral ICANS when ongoing deficits are present but current tools fail to characterise them.

In summary, several useful lessons are suggested from our analysis. First, vigilance for spinal ICANS is required in at‐risk individuals where daily neurological examination to detect emerging myelopathic features is recommended. Second, when spinal ICANS is suspected, MRI cervico‐thoracic spine should be performed as early as possible, as spinal ICANS imaging appearances resolve very quickly with corticosteroids. Finding MRI features associated with spinal ICANS may be diagnostically helpful and save the patient from undergoing extensive additional diagnostic testing. Third, early neurology specialist input is critical given the rarity of spinal ICANS and the wide differential diagnosis for myelopathy following CAR‐T. Neurology input is also important to guide symptom management and rehabilitation. Specialist neuro‐physiotherapist expertise and multidisciplinary care are also usually necessary, particularly to manage neuropathic discomfort, sphincter disturbance and sexual dysfunction, commonly associated with spinal cord injury. Finally, we suggest that resolution of cerebral ICANS symptoms and MRI cord imaging changes could be used to guide corticosteroid taper rather than waiting for clinical resolution of spinal deficits which is frequently extremely slow, analogous to the timelines observed in autoimmune transverse myelitis.

To better understand and manage spinal ICANS, future work will require the integration of neurological phenotyping, MR imaging changes and deeper CSF analysis to determine biomarkers and underlying mechanisms for this unusual, debilitating complication of CD19 CAR‐T therapy.

## AUTHOR CONTRIBUTIONS

FWV, ASC and CR conceived the idea. FWV wrote the first draft of the manuscript. HH, MR, ASC and CR provided critical review. FWV, IM, ASC and CR developed the manuscript revisions. FWV developed Figure 1. FWV, IM, SK, NG, SA, SK, SM, SK, BU, KP, MR and CR collected and analysed the clinical data. HH analysed imaging data and developed Figure 2. All authors read and provided critical input and revisions for the final manuscript.

## FUNDING INFORMATION

No formal project funding.

## CONFLICT OF INTEREST STATEMENT

MR has served on advisory boards and received honoraria from Kite/Gilead, Novartis and Janssen. CR has served on advisory boards and/or received honoraria from Kite/Gilead, Novartis, Autolus, Johnson & Johnson, Bristol Myers Squibb, Cellistic and Kyverna. ASC has served on advisory boards and received honoraria from CSL Behring, Takeda, Griffols, Anylnam, Akcea, Bristol Myers Squibb and Astellas. ASC received a stipend from *BMJ* journals for the associate editor role with JNNP. The remaining authors have nothing to disclose.

## ETHICAL APPROVAL

We obtained retrospective ethics approval through the Integrated Research Application System for all UK CAR‐T centres, which can be accessed through https://www.ema.europa.eu/en/cellular‐therapy‐module‐european‐society‐blood‐marrow‐transplantation‐ebmt‐registry.

## PATIENT CONSENT STATEMENT

Ethical approval was based on patients having given informed consent at the time of referral for the collection of minimally identifiable data as per the European Society for Blood and Marrow Transplantation (EBMT) policy. We have carefully checked that the data included in this manuscript do not enable patient identification on an individual level. Individual patient consent was obtained where possible.

## Supporting information


Table S1.

## Data Availability

De‐identified data that support the findings of this study are available on request from the corresponding author.
