# Supplementary material for: Transient imaging changes accompany ‘spinal ICANS’ following CAR T‐cell therapy for large B‐cell lymphoma in adults
Source: Br J Haematol. 2025 Oct 13;207(6):2632–7. doi: 10.1111/bjh.70206 (PMC12710107; doi:10.1111/bjh.70206)
Supplement: Supplementary file 1 — Table S1. [file BJH-207-2632-s001.docx]

**Supplementary Data**

**Patient 1:** A 27-year-old right-handed patient was treated with axi-cel for refractory primary mediastinal B-cell lymphoma (PMBCL). On day +3 they developed grade 2 CRS and received 3 doses of tocilizumab over 3 days (Figure 1, Supplementary Table 1). On day +5, they developed grade 4 ICANS with confusion, speech impairment, and later a generalised convulsive seizure.

The patient was admitted to ICU (not ventilated) and loaded with levetiracetam with the subsequent addition of phenytoin due to ongoing seizure activity. Intravenous methylprednisolone (IVMP) (1g for 3 days) and anakinra (100mg twice daily, continued until day +12) were administered for high-grade ICANS with resolution of all seizure activity by the afternoon of D+7.

CSF obtained at the time of ICU admission showed markedly elevated protein of 9.3 g/L (reference range, 0.08 - 0.32 g/L) but findings were otherwise normal, including white cell count < 1/mm^3^ and negative viral polymerase chain reaction (PCR) which tests for a wide range of viruses. Serum phosphate was low at 0.59 mmol/L (reference range 0.8 - 1.5 mmol/L)*,* which may have been secondary to seizure activity or a marker of severe ICANS and normalised with replacement over the subsequent 6 days.

On day +7, whilst seizure activity was well controlled and the patient was alert (but not orientated), the immune effector cell-associated encephalopathy (ICE) score remained low at 0 and the patient was noted to have new onset bilateral leg weakness. By day +9, the ICE score had improved to 9/9, but the bilateral leg weakness had evolved into complete flaccid paralysis (MRC grade 0 in all muscle groups) with upgoing plantar reflexes and sphincter dysfunction without a detectable sensory level. Magnetic resonance imaging (MRI) brain and cervical spine showed T2/fluid attenuated inversion recovery (FLAIR) hyperintensity with restricted diffusion in the bilateral hippocampi, periventricular region with continuity to corticospinal tracts over multiple spinal segments (Figure 2, panel A). Clinical and radiological findings suggested an atypical manifestation of ICANS. An infective cause was unlikely given acellular CSF and imaging was inconsistent with spinal cord infarction. A repeat MRI on day +11 showed ongoing widespread periventricular signal change and diffuse T2/FLAIR hyperintense signal extending the length of the spinal cord.

By day +16 leg weakness showed some improvement (hip flexion 4+/5 right, 4/5 left, knee flexion 3/5 right, 4-/5 left, knee extension 4-/5 right, 4/5 left), but the patient remained weak in a pyramidal pattern (flexors weaker than extensors, consistent with an upper motor neuron deficit), with ongoing sphincter dysfunction. A third MRI on day +17 showed minor improvement in periventricular signal change and stable appearances elsewhere (Figure 2, panel B). Levetiracetam was continued but phenytoin was stopped by day +20. Steroids were weaned slowly and stopped on day +26. Clinically, the lower limb weakness continued to improve, and the patient could mobilise with one stick at discharge on day +32. At month 3, power had returned to 5/5 bilaterally in the lower limbs, but a PET-CT demonstrated progressive disease (PD) and the patient died shortly after.

**Patient 2**: A right-handed 61-year-old patient was treated with axi-cel for large B-cell lymphoma (LBCL). CRS developed on day 0, progressing to grade 2 by day +1 and requiring 2 doses of tocilizumab (Figure 1, Supplementary Table 1). On day +3 the patient developed expressive dysphasia, progressing to global aphasia and a seizure, with reduction in Glasgow Coma Score (GCS) to 6/15 (ICANS grade 4). CT head was normal. The patient was sedated, intubated, ventilated and received dexamethasone, tocilizumab, and levetiracetam for seizures. Anakinra (800mg per day) was added on day +5. Blood tests revealed a phosphate of 0.42 mmol/L. CSF analysis on day +5 showed raised protein of 6.95 g/L, a normal white cell count (lymphocytes 1/mm^3^) and negative viral PCR. During a period of reduced sedation on D+5, lower limb weakness was noted. MRI brain on day +6 showed mild bilateral patchy hyperintensity in the thalami, dorsal pons, medulla and bilateral hippocampi and sagittal FLAIR images with increased signal in the cervical spinal cord. (Figure 2 panel C).

Clinically, seizures stopped on day +10 and the ICE score returned to 10/10 by day +13.

Anakinra and Dexamethasone were stopped on day +19. Clinical examination on day +20 (the first opportunity following extubation) revealed severe lower limb pyramidal pattern weakness (knee flexion 0/5 bilaterally, knee extension 1/5 bilaterally). Sphincter involvement was also noted. The patient described numbness over his legs, although sensory examination was objectively intact to all modalities. Repeat MRI on day +21 showed complete resolution of previous imaging changes in the cervical spine (Figure 2, panel D) but new punctate foci of white matter hyperintensity in the brain, as previous reported in ICANS^4^. Repeat CSF analysis showed normalisation of protein by day +33.

On examination on day +51, lower limb weakness had improved slightly (hip flexion 2/5 bilaterally, hip extension 2+/5, knee flexion 3/5 bilaterally, knee extension 1/5 bilaterally with muscle wasting evident). The patient was discharged on day +55 to an inpatient neurorehabilitation centre with some functional impairment, requiring assistance with activities of daily living (ADLs). Complete metabolic response (CMR) was confirmed at 3 months and was ongoing at month 19 when the patient was walking with minimal support and was independent in all ADLs.

**Patient 3:** A 49-year-old patient with LBCL was treated with axi-cel. Grade 2 CRS occurred on day +1, treated with 1 dose of tocilizumab. Further tocilizumab was administered for refractory CRS on days +2, +3 and +5, with the addition of dexamethasone on day +5 when the patient developed grade 3 ICANS, with aphasia, inability to follow commands, and an ICE score of 0/10 (Figure 1, Supplementary Table 1). CT brain was normal and CSF showed an elevated protein of 4.6 g/L with otherwise normal values. Phosphate was mildly low at 0.78 mmol/L. Treatment was escalated to IVMP (1g/day) and the patient improved rapidly by day +6 with an ICE score of 10/10. However, by day +7 the patient developed new areflexic lower limb paralysis, bowel incontinence with a sensory level to the T10 dermatome. MRI brain and spine showed extensive, symmetrical T2/FLAIR signal hyperintensity throughout both cerebral hemispheres continuous with central cord hyperintensity throughout the entire spinal cord (Figure 2, panel E). The syndrome was diagnosed as an atypical manifestation of ICANS as investigations excluded other causes. IVMP was given for a total of 5 days (days +5 to +9 with slow weaning thereafter) alongside Anakinra from day +8 until day +14. Limited clinical response was observed by day +15 and 5 days of 2g/kg intravenous immunoglobulin (IVIg) was administered, again without immediately evident clinical benefit. Repeat MRI on day +24 showed near complete resolution of the extensive signal change throughout the brain and spinal cord, with no new lesions (Figure 2, panel F). Repeat CSF protein was markedly improved from day +5, albeit levels were still borderline elevated (0.55 g/L).

Clinically, the patient remained very weak (hip flexion/extension 1/5 bilaterally, ankle dorsiflexion and plantarflexion both 2/5 bilaterally) and was transferred for inpatient neurorehabilitation on day +44. Month 3 PET-CT showed CMR and the patient was finally discharged home 5 months post-CAR-T with incomplete neurological recovery (hip flexion 4/5, knee extension/flexion 3/5, and ankle dorsiflexion/plantarflexion 3/5 bilaterally). Despite weakness, the patient demonstrated improved function including the ability to lie and sit independently and the ability to sit to stand with sara-steady and self-propel to over 100m using a Zimmer frame at 6 months then crutches at 9 months.

**Patient 4:** A 22-year-old patient received axi-cel for primary refractory LBCL. On day +2, they developed grade 2 CRS, requiring 1 dose of tocilizumab (Figure 1, Supplementary Table 1). Grade 4 ICANS followed on day +5 with status epilepticus on day+6. The patient was intubated/ventilated, treated with levetiracetam, phenytoin and IVMP 1g/day for 3 days. MRI brain/spine on day +7 demonstrated diffuse T2W hyperintensity within the white matter tracts of the brain and whole cord (Figure 2, panel G). CSF on day +8 showed elevated protein of 2.79g/L (reference range, 0.08 - 0.32 g/L) but was otherwise normal, including normal white cell count and negative viral PCR. On day +10, shortly after extubation, lower limb flaccid paralysis with upgoing plantars, sphincter dysfunction and preserved sensation was noted. Serum phosphate was low (0.26 mmol/L). Clinically on day +12, legs remained weak (hip flexion 2/5, knee flexion/extension 2/5) but MRI spine showed significant resolution of signal change, with some residual hyperintensity (Figure 2, panel H), and MRI brain was normal. By discharge on day +40, the lower limb weakness had incompletely recovered, and the patient required ongoing assistance with mobilisation. Sphincter dysfunction resolved at week 6. At month 3, PET-CT showed CMR and by month 6 complete neurological recovery was confirmed.

**Supplementary Table 1:** Pre- and post-infusion clinical, radiographic, and treatment data. ADL: Activities of daily living; CAR-T: chimeric antigen receptor T-cells; CRS: cytokine release syndrome; DLBCL: diffuse large B-cell lymphoma; cells/µL: cells per microlitre; CSF: cerebrospinal fluid; CMR: complete metabolic response; FLAIR: fluid-attenuated inversion recovery; Flu/Cy: fludarabine/cytarabine; g/L: grams per litre; ICANS: immune effector cell-associated neurotoxicity syndrome; IVIg: intravenous immunoglobulin; MRI: magnetic resonance imaging; mmol/L: millimoles per litre; PCR: Polymerase Chain Reaction; PMBCL: primary mediastinal B-cell lymphoma. Normal range for serum phosphate 0.8 - 1.5 mmol/L. Normal range for CSF protein 0.08 - 0.32 g/L.

|  | **Variable** | **Patient 1** | **Patient 2** | **Patient 3** | **Patient 4** |
| --- | --- | --- | --- | --- | --- |
| Baseline Characteristics | **Age at Infusion** | 27 | 61 | 49 | 22 |
|  | **Sex** | Female | Male | Female | Female |
|  | **Cancer Diagnosis** | PMBCL | DLBCL | DLBCL | DLBCL |
|  | **CAR T Product** | Axi-cel | Axi-cel | Axi-cel | Axi-cel |
|  | **Lymphodepletion Regimen** | Flu/Cy | Flu/Cy | Flu/Cy | Flu/Cy |
| Acute Clinical Course | **Limb weakness onset, days after infusion** | Day 7 | Day 5 | Day 7 | Day 10 |
|  | **Acute Examination Findings** | Lower limb weakness, upgoing plantars. Latterly sphincter dysfunction | Pyramidal pattern lower limb weakness. Latterly subjective lower limb sensory loss | No movement in lower limbs bilaterally. Reduced sensation up to chest | Lower limb weakness, upgoing plantars. Latterly urinary incontinence |
| Investigations | **Serum phosphate** | 0.59 mmol/L | 0.42 mmol/L | 0.78 mmol/L | 0.26 mmol/L |
|  | **CSF White Cell Count (day obtained)** | <1 cells/µL (D+5) | 1 cell/µL (D+4) | <1 cells/µL (D+6) | <1 cells/µL (D+8) |
|  | **CSF Protein** | 9.3 g/L | 6.95 g/L | 4.6 g/L | 2.79g/L |
|  | **CSF Viral PCR** | Negative | Negative | Negative | Negative |
|  | **Pertinent MRI brain findings (day obtained)** | T2/FLAIR hyperintensity with restricted diffusion in bilateral hippocampi, olivary nuclei, periventricular region and corticospinal tracts (D+7). | Mild bilateral patchy hyperintensity in the thalami, dorsal pons, medulla and bilateral hippocampi, possibly related to seizure activity (D+6) | Extensive, symmetrical signal hyperintensity throughout both posterior frontal, parietal and medial temporal lobes (D+7) | T2/FLAIR hyperintensity with restricted diffusion in the hippocampi and olivary nuclei (D+7) |
|  | **Pertinent MRI spine findings (day obtained)** | T2/FLAIR hyperintensity in corticospinal tracts (D+7), with signal change in the central grey matter extending the length of the spinal cord. (D+11). Some residual change still evident on D+17. | Longitudinally extensive signal change. Some circumfrential contrast enhancement (D+6) with resolution by D+21 | Longitudinally extensive central cord hyperintensity (D+7). Complete resolution by D+24 | Signal change in central grey matter of spine (D+7). Significant resolution of signal change, with some residual hyperintensity (D+12) |
| Concomitant CRS and ICANS | **Maximum grade CRS** | Grade 2 | Grade 2 | Grade 2 | Grade 2 |
|  | **Maximum grade ICANS** | Grade 4 | Grade 4 | Grade 3 | Grade 4 |
| Therapy Received | **Seizures** | Yes | Yes | No | Yes |
|  | **Tocilizumab** | Yes | Yes | Yes | Yes |
|  | **Anakinra** | Yes | Yes | Yes | No |
|  | **Steroids** | Yes | Yes | Yes | Yes |
|  | **IVIg** | No | No | Yes | No |
| Outcome | **Findings** | Full power had returned at 3 months but patient demonstrated progressive diseases and died shortly afterwards | CMR ongoing at month 19 with patient walking with minimal support and independent in all ADLs | CMR at month 3 but persisting weakness. Patient able to sit to stand with sara-steady and self-propel to over 100m using a Zimmer frame at 6 months then crutches at 9 months. | CMR at month 3 and complete neurological recovery by month 6. |
